# Asymptotic online FWER control for dependent test statistics

**DOI:** 10.1177/09622802261461776

**Published:** 2026-07-02

**Authors:** Vincent Jankovic, Lasse Fischer, Werner Brannath

**Affiliations:** 1 Competence Center for Clinical Trials Bremen, University of Bremen, Bremen, Germany

**Keywords:** asymptotics, dependent test statistics, familywise error rate, online multiple testing, platform trials

## Abstract

In online multiple testing, an a priori unknown number of hypotheses are tested sequentially, that is, at each time point, a test decision for the current hypothesis has to be made using only the data available so far. Although many powerful test procedures have been developed for online error control in recent years, most of them are designed solely for independent or at most locally dependent test statistics. In this work, we provide a new framework for deriving online multiple test procedures that ensure asymptotical (with respect to the sample size) control of the familywise error rate, regardless of the dependence structure between test statistics. In this context, we give a few concrete examples of such test procedures and discuss their properties. Furthermore, we conduct a simulation study in which the type I error control of these test procedures is also confirmed for a finite sample size, and a gain in power is indicated.

## 1. Introduction

In times when ever larger amounts of data become more readily available, there is a growing interest in carrying out numerous statistical tests as quickly as possible. To this end, the online setting that was introduced by Foster and Stine^1^ provides a flexible framework where it is assumed that an unknown and arbitrarily large number of hypotheses can be tested in a sequential manner. In order to address the arising multiplicity problems, many different online test procedures have been developed over the years. While most of them aim for control of the false discovery rate,^1–4^ there are also situations in which the control of the familywise error rate (FWER) may be more appropriate.

This can be the case when optimizing machine learning algorithms^5^ or in a platform trial, where different treatment arms start at different time points and use a shared control group.^6^ Tian and Ramdas^7^ developed online procedures specifically for FWER control, for example, the powerful ADDIS (Adaptive-Discarding) spending algorithm. Furthermore, Fischer et al.^8^ derived an online version of the traditional closure principle, and the application and interpretation of ADDIS procedures were facilitated using graphical approaches.^9^

Despite the great progress in the online literature in recent years, there is still a lack of methods that work for dependent test statistics or p -values.^10^ However, in reality, it is often much more plausible that there are certain underlying dependencies.

**Figure 1.**
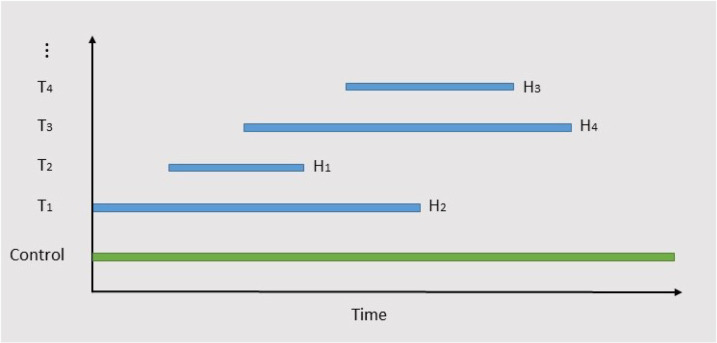
Diagram of a platform trial.

As a concrete motivational example, consider the case of a platform trial (see Figure 1). Here, different treatments may enter the trial at any time. Each treatment arm is tested against the control once sufficient data have been collected for that arm. This results in a sequence of hypothesis tests, with their order determined by when each treatment arm accrues sufficient data for analysis. In this situation, dependency issues arise due to the shared control data. Online algorithms for error control in platform trials have been considered, for example, by Robertson et al.^6^ and Zehetmayer et al.^11^

But there are also other reasons why observations corresponding to different hypotheses could be highly dependent. For example, the observations regarding different hypotheses could originate from the same individual, or a hypothesis that was not rejected at first could be tested again using old and new data. Another application that causes dependencies would be public databases, where different independent research groups have access to the same data and perform their own tests and analyses.^10^

In the literature so far, the test levels of online procedures depend only on the previous p -values or discrete transformations of them.^3,4,7^ When p -values are dependent, it is no longer possible to use them directly for specifying test levels. Zrnic et al.^12^ circumvented this issue by using only the information of p -values that are guaranteed to be independent from the current one, which requires some knowledge of the dependency structure. In this paper we propose a flexible method, that uses the data (instead of just the p -value) and can be equally applied, even when there are dependencies in the data and no or few information about the exact dependency structure is available. Thereby we make use of a resampling method that is known as the low intensity bootstrap (also referred to as m out of n bootstrap) originally proposed by Swanepoel^13^ and thoroughly discussed by Bickel et al.^14^ This is a resampling strategy, where resampling is done with a lower sample size ( m instead of n ) than in the original sample. It can be shown that, in some cases, this leads to consistency, even when the traditional bootstrap fails.^14^

The remainder of the paper is structured as follows: in Section 2 we introduce the underlying set-up and notation and review some of the already existing methods for online FWER control. The main results are given in Section 3, where we introduce the notion of consistent weights and, building upon this, derive new online procedures for FWER control under dependency. In Section 4 numerical simulations are performed in two different settings to exemplify the theoretical considerations. The results are again summarized and discussed in Section 5. Additional technical proofs, detailed derivations, and further simulation results are provided in the Supplementary Material.

## 2. Preliminaries

### 2.1. Notation and model set-up

Suppose we want to sequentially test null hypotheses $H_{1},H_{2},H_{3},\ldots$ and at each step i we perform the test based on data $X_{i}$ , generated by the true distribution P . In each case, the corresponding sample size, denoted by $n_{i}$ , is assumed to be data independent. We make no further assumptions on the data sets, so they possibly could have an arbitrary overlap for different hypotheses, or even coincide. With n∈N , we denote a general parameter of the sample size, that is, for all i∈N , it holds that $n_{i}\to \infty$ as n→∞ . All asymptotic considerations throughout this paper are performed by letting n→∞ . When testing the null hypothesis $H_{i}$ versus the alternative $H_{i}^{\prime }$ , the decisions are made in terms of test statistics $T_{i,n}(X_{i})$ or p -values $P_{i,n}(X_{i})$ . The latter is more in line with the common assumption in the online multiple testing literature. For ease of notation, we typically just use the index n to denote sample size dependent quantities. For instance, we write $P_{i,n}$ instead of $P_{i,n_{i}}$ . An *online test procedure* is then a family of test levels $(\alpha_{i,n}{)}_{i\in N}$ that depend on the previous data, that is, $\alpha_{i,n}=\alpha_{i,n}(X_{1},\ldots ,X_{i-1})$ . We will not use the more general mathematical definition of an online multiple test suggested by Fischer et al.^8^

For an N∈N , let further denote the set of indices corresponding to the true null hypotheses among $H_{1},\ldots ,H_{N}$ by $H^{0}(N)$ and the number of erroneously rejected hypotheses by $V_{n}(N)=\sum_{i\in H^{0}\left(N\right)}1\{P_{i,n}\leq \alpha_{i,n}\}$ . Then, the FWER is defined as

$${\mathrm{FWER}}_{n}(N)=P(V_{n}\left(N\right)\geq 1).$$

The goal is to derive online test procedures that control the FWER asymptotically, that is, for any N∈N , it holds that

(1) $$\underset{n\to \infty }{lim sup}{\mathrm{FWER}}_{n}(N)\leq \alpha .$$

Equation (1) says that the FWER is controlled asymptotically (with respect to the sample size) for any finite number of hypotheses N . Unlike the case of exact error control, where this would also imply the statement for an infinite number of hypotheses, we can *not* guarantee that

$$\underset{n\to \infty }{lim sup}\underset{N\to \infty }{lim sup}{\mathrm{FWER}}_{n}(N)\leq \alpha$$

will hold. For this, further assumptions regarding uniform convergence among all hypotheses might be needed, which are not realistic in this general hypothesis testing set-up. Moreover, (1) reflects the common situation found in essentially all real-life applications, where the number of hypotheses is a priori unknown but finite. Asymptotic control of the FWER implies that the nominal level is expected to be closely maintained in practice, provided the sample size is sufficiently large. This notion is frequently discussed in the literature, particularly in the context of resampling methods.^15,16^ Similar to other asymptotic results commonly relied upon in practice – most notably the central limit theorem – it is challenging to establish a universally valid threshold for what constitutes a “large enough” sample size. Therefore, it is essential to complement theoretical results with simulation studies to assess performance in finite samples.

### 2.2. Existing procedures for online familywise error rate control

We begin by reviewing some of the currently existing procedures for online FWER control. The most straightforward method is the so-called *Alpha-Spending* algorithm.^1^ The idea is to simply divide the global error budget among all hypotheses, and thus it can be seen as an online version of the weighted Bonferroni correction in traditional multiple testing. This can be implemented by selecting a non-negative sequence $(\alpha_{i}{)}_{i\in N}$ such that

$$\sum_{i\in N}\alpha_{i}=\alpha .$$

It can easily be shown by the Bonferroni inequality that this procedure controls the FWER, regardless of the dependence structure of the p -values. Unfortunately, it is generally conservative, so there is an interest in developing new and more powerful online multiple testing procedures.

Tian and Ramdas^7^ derived advanced spending algorithms, most notably the powerful *ADDIS-Spending* procedure, which combines the concepts of adaptivity and discarding. Since we focus in this work on the notion of adaptivity (see also the discussion in Section 5), that is, accounting for the proportion of true null hypotheses, we briefly introduce the so called *Adaptive-Spending*.^7^ For this, let λ∈(0,1) , and we define the so called *candidate indicator* of the i -th p -value by $C_{i}=1\{P_{i}\leq \lambda \}$ . Tian and Ramdas showed that, as long as the p -values are independent, it is sufficient for FWER control that the online procedure $(\alpha_{i}{)}_{i\in N}$ satisfies

(2) $$\sum_{i\in N}\frac{1-C_{i}}{1-\lambda }\alpha_{i}\leq \alpha .$$

Here, the local test levels $\alpha_{i}$ are allowed to depend on the indicators $C_{1},\ldots ,C_{i-1}$ but not on $C_{i}$ .

As a concrete example of such a procedure, they proposed to pick a non-negative sequence $(\gamma_{i}{)}_{i\in N}$ such that $\sum_{i\in N}\gamma_{i}=1$ and define the test levels as

(3) $$\alpha_{i}=\alpha (1-\lambda )\gamma_{t(i)},$$

where $t(i)=1+\sum_{j<i}(1-C_{j})$ .

The idea of (2) is to decrease the remaining error budget only when a true null hypothesis has been tested. The use of the candidate indicator, as a surrogate for the indicator of a true alternative, relies on the fact that p -values corresponding to true alternatives are usually small and therefore rather unlikely to exceed the threshold λ . The factor (1−λ) accounts for the p -values, for which the null hypothesis is true (from now on referred to as *null p -values*), that are by chance smaller than λ .

Since the test levels in (2) depend directly on the previous p -values, we need the underlying assumption that the null p -values are valid conditional on the previous p -values, that is, for all $i\in H^{0}\left(N\right)$ and x∈[0,1] , it holds that

(4) $$P(P_{i}\leq x|P_{1},\ldots ,P_{i-1})\leq x.$$

Using the same proof as Tian and Ramdas,^7^ it is clear that FWER control is guaranteed when (2) and (4) are fulfilled.

While (4) is always true when the p -values are independent, it is easily violated when dependencies between the p -values are present, as demonstrated in the following example.

Example 2.1 Let $\left(Z_{1},Z_{2}\right)$ be bivariate normally distributed with means $E(Z_{1})=E(Z_{2})=0$ , variances $\mathrm{var}(Z_{1})=\mathrm{var}(Z_{2})=1$ and covariance $\mathrm{cov}(Z_{1},Z_{2})=\rho >0$ . Consider the one-sided p -values $P_{i}=1-\Phi (Z_{i})$ for i=1,2 . With $c_{1}<1/2<c_{2}$ and $q_{i}=\Phi^{-1}(1-c_{i}),i=1,2$ , it holds that

$$\begin{array}{rl} & P(P_{2}\leq c_{2}|P_{1}=c_{1})\\ & \;=P(Z_{2}\geq q_{2}|Z_{1}=q_{1})\\ & \;=P\left\{(Z_{2}-\rho q_{1})/(1-\rho^{2}{)}^{1/2}\geq (q_{2}-\rho q_{1})/(1-\rho^{2}{)}^{1/2}|Z_{1}=q_{1}\right\}\\ & \;=1-\Phi \left\{(q_{2}-\rho q_{1})/(1-\rho^{2}{)}^{1/2}\right\}\\ & \;>1-\Phi (q_{2})=c_{2}. \end{array}$$

In the penultimate line it is used that the conditional distribution of $Z_{2}$ given $Z_{1}=q_{1}$ is normal with mean $\rho q_{1}$ and variance $1-\rho^{2}$ . Moreover, the final inequality holds, since $q_{2}<0$ , $\rho q_{1}>0$ , and $(1-\rho^{2}{)}^{1/2}<1$ according to the assumptions.

## 3. Multiple testing with consistent weights

### 3.1. Consistent weights

As discussed in Section 2.2, the main idea of the Adaptive-Spending algorithm is to categorize the p -values based on whether they are believed to have arisen under the null hypothesis or the alternative. This is achieved by using information from the indicators, $C_{i}$ , which are discrete transformations of the p -values, when setting the test levels. Because of that, the p -value $P_{i}$ and the corresponding test level $\alpha_{i}$ are dependent, unless the p -values themselves are independent. In general, this dependency between the p -values and the corresponding test level causes the Adaptive-Spending procedures to fail, that is, error control can no longer be guaranteed. To address this issue, we aim to generate test levels that use information from the data but become deterministic as the sample size tends to infinity. By this, every p -value and its corresponding test level are asymptotically independent. Therefore, we introduce the notion of a weight $\xi_{i,n}=\xi_{i,n}(X_{i})$ that captures, in a sense, the likelihood of a p -value being a null p -value and replaces the term $1-C_{i}$ in Adaptive-Spending algorithms. We now state the formal general property of these weights.

Definition 3.1 **(Consistent weights)**

Let $\lambda_{i}\in (0,1)$ for every i∈N . We call an array of random variables $(\xi_{i,n}{)}_{i\in N,n\in N}$ with values in [0,1] an array of *consistent weights* if there exist constants $c_{i}\in [0,1],i\in N$ , with $c_{i}\geq 1-\lambda_{i}$ for $i\in H^{0}:=\underset{N\in N}{⋃}H^{0}(N)$ such that

(5) $$\xi_{i,n}\to c_{i},\;(n\to \infty )$$

in probability for every i∈N .

Although not required for FWER control, the weights $\xi_{i,n}$ should ideally also be close to 0 when the alternative $H_{i}^{\prime }$ is true. For example, defining $\xi_{i,n}=1-\lambda_{i}$ for all n∈N would obviously satisfy (5), but it would not help identify non-null p -values. Note, for example, that neither the p -values $P_{i,n}$ nor the expressions $1-C_{i,n}=1\{P_{i,n}>\lambda_{i}\}$ satisfy assumption (5), since under the exact null hypothesis $P_{i,n}$ is uniformly distributed on [0,1] for any n∈N .

Next, we illustrate two methods for obtaining weights that satisfy (5) from observed data.

Example 3.2 Assume that, in each step i , the observed data $X_{i}$ consist of random variables $X_{i,1},\ldots ,X_{i,n_{i}}∼N(\mu_{i},1)$ , and we want to test the hypothesis $H_{i}:\mu_{i}\leq 0$ versus the alternative $H_{i}^{\prime }:\mu_{i}>0$ . The test statistic and the corresponding one-sided p -value are

$$Z_{i,n_{i}}=n_{i}^{1/2}{\bar{X}}_{i,n_{i}},\;\;\;P_{i,n_{i}}=1-\Phi (Z_{i,n_{i}}),$$

where ${\bar{X}}_{i,n_{i}}$ is the sample mean and Φ denotes the cumulative distribution function of the standard normal distribution. Furthermore, let $(a_{n}{)}_{n\in N}$ be a non-decreasing sequence of positive real numbers that diverges to infinity. For any $\lambda_{i}\in (0,1)$ we define consistent weights as

$$\xi_{i,n_{i}}=\left\{ \begin{array}{ll} 0 & \text{ if }Z_{i,n_{i}}>a_{n_{i}}\\ 1-\lambda_{i} & \text{ if }Z_{i,n_{i}}\leq a_{n_{i}} \end{array}\right..$$

It is straightforward to verify that these weights satisfy the conditions in Definition 3.1. Although this method is simple to implement, one drawback is that the sequence $(a_{n}{)}_{n\in N}$ lacks a direct interpretation, which makes choosing appropriately difficult. Additionally, it is important to note that these weights are systematically biased in the mean towards 0 under the null hypothesis, which can result in rather anti-conservative procedures for finite sample sizes.

For a more advanced example, we use a resampling technique that is referred to as the low intensity bootstrap. The idea is to resample fewer observations than in the original sample to remedy consistency issues. A commonly given heuristic motivation is that resampling with fewer observations more accurately reflects the relationship between the original sample and the entire population.^17^ The specific resampling scheme depends on the form of the data, $X_{i}$ , and the test to be conducted. In the following example, which was given in a similar form by Neumann et al.,^18^ we illustrate the procedure for the parametric one-sample test situation, as in Example 3.2. The method can be adapted to, for example, a non-parametric setting^17^ or 2-sample tests.

Example 3.3 **(Low intensity bootstrap example)**

We are assuming the same set-up and notation as in Example 3.2. Additionally, consider the bootstrap variables $X_{i,1}^{*},\ldots ,X_{i,m(n_{i})}^{*}$ resulting from a parametric bootstrap of size $m(n_{i})$ , that is, they are independently drawn from the distribution $N({\bar{X}}_{i,n_{i}},1)$ . Here, m:N→N is a non-decreasing map such that m(n)→∞ and m(n)/n→0 as n→∞ . Additionally, we construct the (low intensity) bootstrap test statistic $Z_{i,m(n_{i})}^{*}=m(n_{i}{)}^{-1/2}\sum_{j=1}^{m(n_{i})}X_{i,j}^{*}$ , whose conditional distribution is a normal distribution with mean $\{m(n_{i})/n_{i}{\}}^{1/2}Z_{i,n_{i}}$ and variance 1. Moreover, we define the low intensity bootstrap version of the p -value $P_{i,n_{i}}$ by $P_{i,m(n_{i})}^{*}=1-\Phi \{Z_{i,m(n_{i})}^{*}\}$ . For any $\lambda_{i}\in (0,1)$ , it holds that

$$\begin{array}{rl} P\{P_{i,m(n_{i})}^{*}>\lambda_{i}|X_{i}\} & =P\{Z_{i,m(n_{i})}^{*}<\Phi^{-1}(1-\lambda_{i})|X_{i}\}\\ & =\Phi \left[\Phi^{-1}(1-\lambda_{i})-\{m(n_{i})/n_{i}{\}}^{1/2}Z_{i,n_{i}}\right]\\ & \;\to \left\{ \begin{array}{ll} 0 & \text{ if }\mu_{i}>0\\ 1 & \text{ if }\mu_{i}<0\\ 1-\lambda_{i} & \text{ if }\mu_{i}=0 \end{array}\right.. \end{array}$$

Thus, by selecting $\xi_{i,n_{i}}=P\{P_{i,m(n_{i})}^{*}>\lambda_{i}|X_{i}\}$ , property (5) is fulfilled. The low intensity bootstrap, that is, a bootstrap sample of size m(n)<n , is necessary because, when the exact null hypothesis $\mu_{i}=0$ is true, $Z_{i,n_{i}}$ is a standard normal random variable regardless of the sample size, and thus, the asymptotic consistency would not be met if $m(n_{i})=n_{i}$ . This issue arises because the bootstrap test statistics are not centralized, as we are not interested in the distribution under the null hypothesis but the true underlying distribution.

As just demonstrated, it is possible to obtain weights $\xi_{i,n}$ from the data by using a low intensity resampling strategy. A common default choice for the size of the resamples is $m(n)=\lfloor n^{1/2}\rfloor$ .^18^ More sophisticated rules regarding the choice of m for the low intensity bootstrap are discussed by Bickel and Sakov.^19^

As mentioned before, besides fulfilling the assumption (5) it is also desirable that $\xi_{i,n}$ tends to 0 under the alternative, which is the case in Example 3.2 and Example 3.3.

Remark 3.4 Although both of the mentioned methods lead to consistent weights and therefore are valid choices for the upcoming procedures, we propose to use the bootstrap weights as in Example 3.3, because they rely less strictly on pure asymptotics compared to the thresholding weights in Example 3.2 and thus seem to perform better in practice. In support of this, it can be shown that for certain situations, procedures using bootstrap weights are guaranteed to have error control not just asymptotically, but also for finite sample sizes (see Theorem 3.11).

### 3.2. Main result

We now present a general framework for incorporating the notions from the previous section into existing algorithms. Therefore, we assume that random variables $\xi_{i,n}$ with property (5) are at hand; for example, they could have been generated according to the low intensity resampling scheme in Example 3.3.

We can now formulate a general rule, very similar to (2), for creating online procedures under dependency: let $\lambda_{i}\in (0,1)$ for every i∈N . Then, test levels $(\alpha_{i,n}{)}_{i\in N}$ have to be chosen in a way such that for every n,N∈N , it holds that

(6) $$\sum_{i=1}^{N}\frac{\alpha_{i,n}}{1-\lambda_{i}}\xi_{i,n}\leq \alpha .$$

Note that, unlike the discrete indicators in (2), the weights $\xi_{i,n}$ can take on any value in the range from 0 to 1. Moreover, since we are aiming for asymptotically valid procedures, condition (6) could be relaxed to the effect that it only needs to hold for all but finitely many n∈N , but this has no real practical relevance. In the next two lemmas, we will show that under mild additional assumptions (see Assumptions 3.5 and 3.6), it is sufficient for asymptotical FWER control to select an online procedure that satisfies (6).

Assumption 3.5 The test levels $\alpha_{i,n}=f_{i}(\xi_{1,n},\ldots ,\xi_{i-1,n})$ are deterministic, continuous functions of the consistent weights.

Assumption 3.6 The null p -values are asymptotically valid, that is, for all $i\in H^{0}\left(N\right)$ , there exists a (super-)uniformly distributed random variable $P_{i}$ , that is, $P(P_{i}\leq x)\leq x$ for all x∈[0,1] , such that $P_{i,n}\to P_{i}$ in distribution as n→∞ .

Remark 3.7 Assumption 3.5 implies that, due to the definition of consistent weights (5) and the continuous mapping theorem, for every i≥1 it holds that $\alpha_{i,n}\to \alpha_{i}:=f_{i}(c_{1},\ldots ,c_{i-1})$ in probability as n→∞ , where $c_{1},\ldots ,c_{i-1}$ are the corresponding weight limits. Assumption 3.6 states that a well-behaved limit distribution must exist. This is a standard assumption used for calibrating asymptotically valid tests and is often ensured by limit theorems. It is, for example, also fulfilled in the test situation described in Example 3.3. There, $P_{i}$ follows a uniform distribution on [0,1] if $\mu_{i}=0$ and $P_{i}=1$ almost surely if $\mu_{i}<0$ .

Lemma 3.8 Let $(\alpha_{i,n}{)}_{i\in N}$ be an online procedure with asymptotically deterministic test levels, that is, for every i≥1 , there exists a constant $\alpha_{i}\in [0,1]$ so that $\alpha_{i,n}\to \alpha_{i}$ as n→∞ . Under Assumption 3.6, it holds for every N∈N that

$$\underset{n\to \infty }{lim sup}E\left(\sum_{i\in H^{0}\left(N\right)}1\{P_{i,n}\leq \alpha_{i,n}\}\right)\leq \sum_{i\in H^{0}\left(N\right)}\alpha_{i}.$$

Proof. Let $i\in H^{0}\left(N\right)$ . By Assumption 3.6 we have that $P_{i,n}\to P_{i}$ in distribution as n→∞ , where $P_{i}$ is a (super-)uniformly distributed random variable. Due to Slutsky’s theorem and the assumption of asymptotically deterministic test levels, we have that $P_{i,n}-\alpha_{i,n}\to P_{i}-\alpha_{i}$ in distribution as n→∞ . It follows that

$$\begin{array}{rl} \underset{n\to \infty }{lim sup}P(P_{i,n}\leq \alpha_{i,n}) & =\underset{n\to \infty }{lim sup}P(P_{i,n}-\alpha_{i,n}\leq 0)\\ & \leq P(P_{i}-\alpha_{i}\leq 0)\\ & =P(P_{i}\leq \alpha_{i})\\ & \leq \alpha_{i}, \end{array}$$

where the first inequality holds due to the Portmanteau theorem. The second inequality results from the fact that $P_{i}$ is (super-)uniformly distributed and $\alpha_{i}$ is a constant. Due to the linearity of the expectation and the subadditivity of the lim sup , the entire inequality also applies to the sum.

Lemma 3.9 Let $(\alpha_{i,n}{)}_{i\in N}$ be an online procedure satisfying (6). Under Assumption 3.5, it holds for every N∈N that

$$\sum_{i\in H^{0}\left(N\right)}\alpha_{i}\leq \alpha .$$

Proof. For all n∈N , we have $\sum_{i\in H^{0}\left(N\right)}\frac{\alpha_{i,n}}{1-\lambda_{i}}\xi_{i,n}\leq \alpha$ almost surely. Due to the definition of consistent weights (5) and Assumption 3.5 for every 1≤i≤N , it holds that $\alpha_{i,n}\to \alpha_{i}$ and $\xi_{i,n}\to c_{i}$ in probability as n→∞ . Since $H^{0}(N)$ is finite, it follows that

$$\sum_{i\in H^{0}\left(N\right)}\frac{\alpha_{i,n}}{1-\lambda_{i}}\xi_{i,n}\to \sum_{i\in H^{0}\left(N\right)}\frac{\alpha_{i}}{1-\lambda_{i}}c_{i}\geq \sum_{i\in H^{0}\left(N\right)}\alpha_{i}.$$

Hence, the lemma.

Now, we can combine the previous lemmas to obtain the main result.

Theorem 3.10 Let $(\alpha_{i,n}{)}_{i\in N}$ be an online procedure satisfying (6) and N∈N . Under Assumptions 3.5 and 3.6 it ensures asymptotical control of the FWER, that is,

$$\underset{n\to \infty }{lim sup}{\mathrm{FWER}}_{n}\left(N\right)\leq \alpha .$$

Proof. This is a direct conclusion of Lemma 3.8 and Lemma 3.9 plus the fact that $P(V_{n}\left(N\right)\geq 1)\leq E(V_{n}\left(N\right))$ .

We conclude this section with a finite sample size result for the case of independent test statistics.

Theorem 3.11 Let $H_{1},H_{2},H_{3},\ldots$ be hypotheses concerning the one-sided testing of means and assume that we have (centralized) estimators ${\hat{\theta }}_{i,n_{i}}$ for i≥1, that are independent and normally distributed with expectation 0 and variance $s_{i,n_{i}}^{2}$ under the null hypothesis. The corresponding p -values are given by $P_{i,n_{i}}=1-\Phi ({\hat{\theta }}_{i,n_{i}}/s_{i,n_{i}})$ . For each i≥1 , let ${\hat{\theta }}_{i,m(n_{i})}^{*}$ be bootstrap estimators obtained through a parametric bootstrap procedure using a sample size $m(n_{i})$ , where m(n)→∞ and m(n)/n→0 if n→∞ , so that ${\hat{\theta }}_{i,m(n_{i})}^{*}|X_{i}\;∼N({\hat{\theta }}_{i,n_{i}},s_{i,m(n_{i})}^{2})$ . Furthermore, consider the bootstrap p -values $P_{i,m(n_{i})}^{*}=1-\Phi \{{\hat{\theta }}_{i,m(n_{i})}^{*}/s_{i,m(n_{i})}\}$ and define consistent weights as $\xi_{i,n_{i}}=P\{P_{i,m(n_{i})}^{*}>\lambda_{i}|X_{i}\}$ , where $\lambda_{i}\in [0.5,1)$ for all i≥1 . Then, any online procedure that satisfies (6) with respect to the weights $\xi_{i,n_{i}}$ ensures exact error control, that is, ${\mathrm{FWER}}_{n}\left(N\right)\leq \alpha$ for every n,N∈N .

### 3.3. Test procedures based on consistent weights

In the following, we give a few examples of concrete implementations of online procedures that assure (6) and thus, according to Theorem 3.10, control the FWER.

Example 3.12 For an easy implementation of an online procedure, we choose the test levels as

(7) $$\alpha_{i,n}=\Pi_{i}(1-\lambda_{i})\left(\alpha -\sum_{j<i}\frac{\alpha_{j,n}}{1-\lambda_{j}}\xi_{j,n}\right),$$

with constants $\Pi_{i},\lambda_{i}\in (0,1)$ . It can be shown that the resulting procedure satisfies condition (6). At any step, the procedure allocates a specific fraction of the remaining alpha budget for the current test. This idea, with candidate indicators instead of consistent weights, was originally considered for false discovery rate control,^20^ where the special case of choosing $\Pi_{i}=\Pi \in (0,1)$ was proposed and the relation to a geometric spending sequence was discussed. With this selection, the same proportion of remaining alpha wealth would be spent at any step. This consistent behavior seems reasonable when there is no information on the number of hypotheses that are going to be tested eventually. Another advantage is that there exists a recursion formula only using the information of the previous test. For i≥1 it holds that

(8) $$\alpha_{i+1,n}=\Pi_{i+1}(1-\lambda_{i+1})\frac{\alpha_{i,n}(1-\Pi_{i}\xi_{i,n})}{(1-\lambda_{i})\Pi_{i}}.$$

When using a constant Π∈(0,1) ^20^ and with the common default choice $\lambda_{i}\equiv \lambda \in (0,1)$ , the update rule further simplifies to

$$\alpha_{i+1,n}=\alpha_{i,n}(1-\Pi \xi_{i,n}).$$

Example 3.13 **(Continuous Adaptive-Graph)**

For a more advanced example, we first briefly review the ADDIS-Graph that was proposed by Fischer et al.^9^ for independent or locally dependent p -values. This is an online procedure that combines the ideas of adaptivity and discarding^7^ and the Online-Graph,^7,9^ where sometimes test levels can be redistributed to future hypotheses. To this end, let $(\gamma_{i}{)}_{i\in N}$ and $(g_{j,i}{)}_{i=j+1}^{\infty },j\in N,$ be non-negative sequences that sum to at most 1 and $\tau_{i}\in (0,1],\lambda_{i}\in [0,\tau_{i})$ for all i∈N . The test levels are then defined as

$$\alpha_{i}=(\tau_{i}-\lambda_{i})\left\{\alpha \gamma_{i}+\sum_{j=1}^{i-1}g_{j,i}(C_{j}-S_{j}+1)\frac{\alpha_{j}}{\tau_{j}-\lambda_{j}}\right\},$$

where $C_{j}=1\{P_{j}\leq \lambda_{j}\}$ and $S_{j}=1\{P_{j}\leq \tau_{j}\}$ denote the indicators for a candidate or non-discarding, respectively. The idea is that in certain cases, namely when a p -value is either classified as a candidate ( $C_{j}=1$ ) or discarded ( $S_{j}=0$ ), the test level can be proportionally distributed to the future hypotheses according to the weights $g_{j,i}$ . To adapt this procedure to the new framework, the candidate indicators are substituted by the consistent weights, $\xi_{j,n}$ . Since in this paper we are solely dealing with the concept of adaptivity, we set $\tau_{j}=1$ and thus $S_{j}=1$ for all j∈N , meaning that no p -value is discarded. This results in the so called *continuous Adaptive-Graph*, whose test levels are then defined by

(9) $$\alpha_{i,n}=(1-\lambda_{i})\left\{\alpha \gamma_{i}+\sum_{j=1}^{i-1}g_{j,i}(1-\xi_{j,n})\frac{\alpha_{j,n}}{1-\lambda_{j}}\right\}.$$

It can be shown that the continuous Adaptive-Graph defined in (9) indeed fulfills condition (6). The procedure in Example 3.12 with constant $\Pi_{i}=\Pi \in (0,1)$ is a special case of (9) by selecting the sequence $\gamma_{i}=\Pi (1-\Pi {)}^{i-1}$ .

### 3.4. Continuous-spending procedures

In Section 3.2, we established the general rule (6) that can be used to produce asymptotically valid online test procedures. While this condition is sufficient, there are also other ways to design asymptotically valid online test procedures that do not necessarily fulfill (6). To exemplify this, we now provide an example of a class of online test procedures that can be seen as a natural adaptation of the Adaptive-Spending algorithm (3).

For a given λ∈(0,1) and a non-increasing and continuous function $f:[1,\infty )\to R_{\geq 0}$ with $\int_{1}^{\infty }f(x)dx<\infty$ , the test levels will be assigned as follows:

(10) $$\alpha_{i,n}=\alpha \frac{\left(1-\lambda \right)}{s}f\left(1+\sum_{j<i}\xi_{j,n}\right),$$

where $s=(1-\lambda )f(1)+\int_{1}^{\infty }f(x)dx$ is a constant scaling factor.

This allocation rule states that, in each step, we lose a bit of wealth determined by the fractions $\xi_{i,n}$ . This is similar to (3), but the discrete sequence $(\gamma_{i}{)}_{i\in N}$ and indicator functions $1\{P_{i}\leq \lambda_{i}\}$ are now replaced by a continuous function and the consistent weights, respectively. In this sense, test procedures according to (10) could be referred to as *continuous adaptive-spending procedures*. Note that λ∈(0,1) is now assumed to be the same for all hypotheses. It is easy to see that for a continuous adaptive-spending procedure, equation (6) does not necessarily hold. Let, for example, λ=1/2 and $f(x)=2x^{-4}$ . Since $s=(1-\lambda )f(1)+\int_{1}^{\infty }f(x)dx=5/3$ , the corresponding test levels are $\alpha_{i,n}=(3\alpha /5)(1+\sum_{j<i}\xi_{j,n}{)}^{-4}$ . It follows that the first summand of the left hand side of (6) is equal to $(6\alpha /5)\xi_{1,n}$ . In general, this expression is not smaller than α , as $\xi_{1,n}$ can take on any value between 0 and 1.

Before we prove error control, we state the following lemma, which takes on a similar role as Lemma 3.9 in Section 3.2.

Lemma 3.14 Let λ∈(0,1) , $f:[1,\infty )\to R_{\geq 0}$ be a non-increasing, continuous, integrable function, and $(\alpha_{i,n}{)}_{i\in N}$ be an online procedure with test levels according to (10). Then, under Assumption 3.5, it holds for every N∈N that

$$\sum_{i\in H^{0}\left(N\right)}\alpha_{i}\leq \alpha .$$

Now, we can state the main theorem for formal error control.

Theorem 3.15 Let $(\alpha_{i,n}{)}_{i\in N}$ be an online procedure according to (10). Under the same conditions as in Lemma 3.14 and Assumption 3.6, it holds for every N∈N that

$$\underset{n\to \infty }{lim sup}{\mathrm{FWER}}_{n}\left(N\right)\leq \alpha .$$

Proof. Since the test levels are continuous transformations of the consistent weights, we can apply Lemma 3.8 to obtain

$$\underset{n\to \infty }{lim sup}{\mathrm{FWER}}_{n}\left(N\right)\leq \sum_{i\in H^{0}\left(N\right)}\alpha_{i}.$$

Now, we can use Lemma 3.14 to complete the proof.

To conclude this section, we give a concrete example of an online procedure according to the continuous-spending approach (10).

Example 3.16 **(Interpolation)**

Let $(\gamma_{i}{)}_{i\in N}$ be a non-negative, non-increasing sequence with $\sum_{i\in N}\gamma_{i}=1$ . Now we define

(11) $$f_{\gamma }(x)=\gamma_{\left\lfloor x\right\rfloor }+(x-\left\lfloor x\right\rfloor )(\gamma_{\left\lceil x\right\rceil }-\gamma_{\left\lfloor x\right\rfloor }),$$

where ⌊x⌋ and ⌈x⌉ denote the greatest integer smaller/least integer greater than or equal to x , respectively. The function $f_{\gamma }$ is the linear interpolation of the sequence $(\gamma_{i}{)}_{i\in N}$ , that is, in particular, it is non-increasing, continuous, and $f_{\gamma }(x)=\gamma_{x}$ for all x∈N . The test levels according to (10) are defined as

(12) $$\alpha_{i,n}=\alpha \frac{\left(1-\lambda \right)}{s}f_{\gamma }\left(1+\sum_{j<i}\xi_{j,n}\right),$$

where the scaling factor simplifies to $s=1+\gamma_{1}(1/2-\lambda )$ .

### 3.5. Closed testing procedures

One of the most important concepts for control of the FWER in the classical offline framework is the *closure principle*.^21^ Here, every intersection hypothesis is tested with a local test, and the resulting multiple test rejects an elementary hypothesis $H_{i}$ if and only if every intersection hypothesis that is contained in $H_{i}$ is rejected by the corresponding local test, granting strong control of the FWER. At first glance, this approach does not seem to be suitable for the online setting, since it requires the prior knowledge of all hypotheses at once. However, Fischer et al.^8^ developed an online version of the closure principle, including a predictability condition, which ensures that in each step, only the intersection of hypotheses up to this point needs to be considered. Moreover, they demonstrated how to convert these online closed procedures into corresponding short-cut procedures. In this section, we apply the online closure principle^8^ to obtain uniform improvements of the online procedures from the previous sections. The central idea is that if any hypothesis is rejected, the corresponding test level could be reused for future hypotheses. Depending on the procedure, this can, for instance, be implemented by shifting or distributing the regained test level. The Online-Fallback procedure^7^ can, for example, be seen as a closure of the Alpha-Spending algorithm.

Let $R_{i,n}=1\{P_{i,n}\leq \alpha_{i,n}\}$ be the rejection indicator of the i -th hypothesis. Then, a closed version of the continuous Adaptive-Graph (9) is given by

(13) $$\alpha_{i,n}=(1-\lambda_{i})\left(\alpha \gamma_{i}+\sum_{j=1}^{i-1}g_{j,i}\max\{1-\xi_{j,n},R_{j,n}\}\frac{\alpha_{j,n}}{1-\lambda_{j}}\right).$$

Similarly, we obtain a closed procedure for the continuous-spending approach (10):

(14) $$\alpha_{i,n}=\alpha \frac{\left(1-\lambda \right)}{s}f\left\{1+\sum_{j<i}(1-R_{j,n})\xi_{j,n}\right\},$$

where $s=(1-\lambda )f(1)+\int_{1}^{\infty }f(x)dx$ .

It is apparent that both of these closed procedures are uniform improvements of the original ones, respectively.

## 4. Simulations

### 4.1. General set-up

To investigate the theoretical results for a finite sample size, we perform a simulation study. Here, we consider two different settings. The first one is a general scenario, where test statistics that are close in time are strongly correlated (e.g. due to data reuse), and those which are further apart from each other have a low or close to zero correlation. The second set-up is more specific and mimics the scenario of a platform trial, where different treatment arms enter the trial at different time points and are tested against a shared control group.

In both settings, we explore the closed versions of the proposed continuous Adaptive-Graph (13) and the continuous adaptive-spending approach (14) (in this section referred to as *Continuous-Graph* and *Continuous-Spending*, respectively), as well as the Online-Fallback procedure,^7^ that grants FWER control regardless of the dependency structure, for comparison. Furthermore, we include the original Adaptive-Spending (3), for which no online FWER control is guaranteed in these scenarios, to evaluate the impact on the power caused by the adjustments for dependency. In all of the following simulations, we choose $g_{j,i}=\gamma_{i-j}$ , $f=f_{\gamma }$ as defined in (11), and set the remaining hyperparameters as λ=1/2 and $\gamma_{j}=6(\pi j{)}^{-2}$ . The consistent weights are defined based on the approach in Example 3.3, as they demonstrated better performance in error control compared to the weights in Example 3.2. The power is defined as the expected proportion of truly rejected null hypotheses out of all false null hypotheses. Throughout, we perform 20000 independent simulation runs to estimate the FWER and power.

### 4.2. Autocorrelation

We test in a Gaussian setting N null hypotheses of the form $H_{i}:\mu_{i}\leq 0$ versus the corresponding alternative $H_{i}^{\prime }:\mu_{i}>0$ . In every run, the parameters $\mu_{i}$ are set according to the following:

$$\mu_{i}=\left\{ \begin{array}{ll} 0 & \text{ with probability }1-\pi_{1}\\ 5 & \text{ with probability }\pi_{1} \end{array}\right.,$$

where $\pi_{1}\in [0,1]$ is the proportion of false null hypotheses. After setting the parameters, we simulate Z -Scores according to a multivariate normal distribution, that is, $\left(Z_{1},\ldots ,Z_{N})∼N(\mu ,\Sigma \right)$ where $\mu =(\mu_{1},\ldots ,\mu_{N})$ is the vector of true effects and $\Sigma =(\Sigma_{ij}{)}_{i,j}\in R^{N\times N}$ is a covariance matrix. We investigate the plausible case where test statistics that are close in time are strongly correlated and the correlation decays rather quickly when test statistics are further apart. Therefore, we set the covariance matrix as

$$\Sigma_{ij}=\left\{ \begin{array}{ll} 1 & \text{ if }i=j\\ \rho^{|i-j|} & \text{ if }i\neq j \end{array}\right.$$

with a fixed ρ∈[0,1] . The weights $\xi_{i,n}$ required for the Continuous-Graph and the Continuous-Spending are constructed by a low intensity bootstrap as in Example 3.3 based on the assumed sample size n . Due to the parametric set-up, the exact bootstrap probabilities $P\{P_{i,m(n)}^{*}>\lambda |X_{i}\}$ can be calculated using only the shrunk version of $Z_{i}$ and the cumulative distribution function of the standard normal distribution, so that no actual resampling is needed. Unless stated otherwise, we set n=100 for the sample size, while varying the proportion of true alternatives $\pi_{1}$ . In additional simulations, we fix $\pi_{1}=0.1$ , and investigate different values of the correlation strength, ρ , or different combinations of the number of hypotheses and the theoretical sample size. In the latter case, we scale down the effect size with respect to the sample size so that the power remains comparable. Moreover, to evaluate the performance of the methods for asymmetric data distributions, we examine the situation where Z -scores are simulated based on the skew-normal distribution^22^ with a skewness parameter of 0.5 .

**Figure 2.**
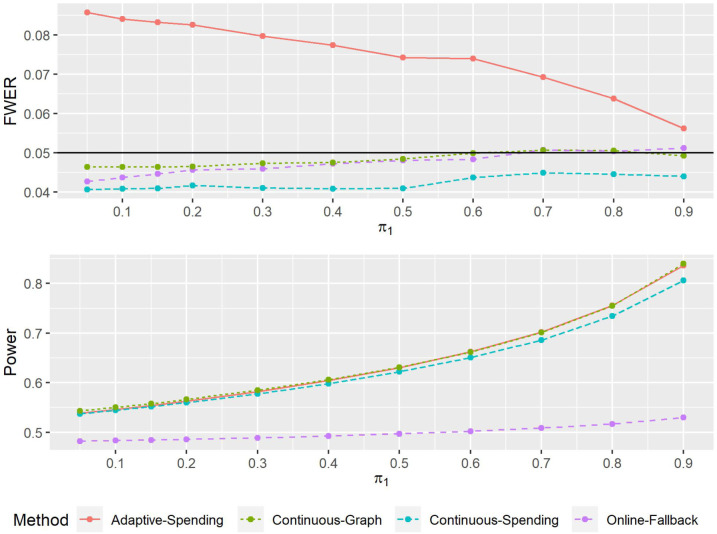
Estimated FWER and power in an autocorrelation set-up with N=1000 , ρ=0.8 and target level α=0.05 plotted over the proportion of false null hypotheses $\pi_{1}$ .

**Figure 3.**
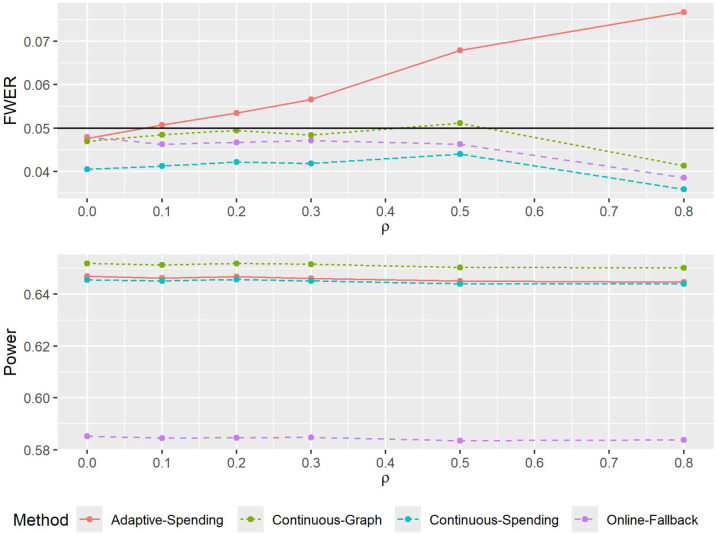
Estimated FWER and power in an autocorrelation set-up with N=500 , $\pi_{1}=0.1$ and target level α=0.05 for different values of ρ .

**Figure 4.**
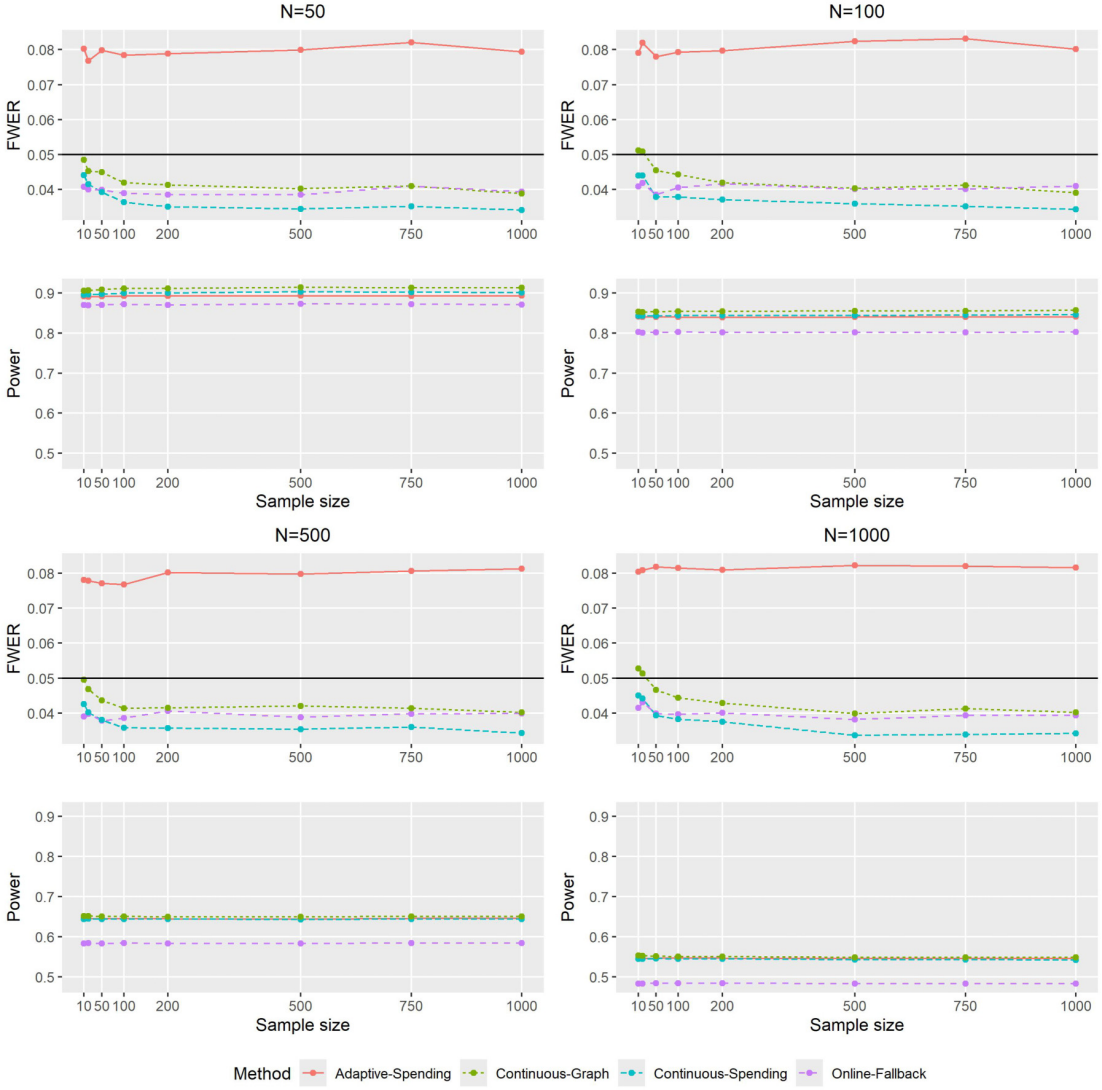
Estimated FWER and power in an autocorrelation set-up with ρ=0.8 , $\pi_{1}=0.1$ and target level α=0.05 for N∈{50,100,500,1000} hypotheses plotted over the sample size.

It can be seen in Figure 2 that the Continuous-Spending, Continuous-Graph, and Online-Fallback seem to control the FWER at the nominal level α . Only the Adaptive-Spending shows a substantial inflation of 60% when the proportion of false null hypotheses is small, which gradually decreases to 20% as the proportion of false null hypotheses increases. In terms of power, it is apparent that the Online-Fallback procedure performs considerably worse than the other three procedures, which is no surprise, as it is the only procedure without a proper built-in mechanism for adaption to the number of true null hypotheses. Here, the Continuous-Graph and the Adaptive-Spending are virtually indistinguishable, while both seem to be slightly more powerful than the Continuous-Spending, where the difference becomes clearer for larger values of $\pi_{1}$ . As can be seen in Figure 3, the FWER inflation of Adaptive-Spending increases, as the strength of the correlation between the p -values increases. For the other methods, the FWER does not appear to be greatly affected by the correlation, and may even decrease slightly for large values of ρ . The power of all methods appears to remain nearly constant, whereas the power of Online-Fallback is consistently lower than that of the other methods. In Figure 4, we observe that the proposed methods control the FWER even for small sample sizes. A slight violation of the nominal level occurs only for the Continuous-Graph at n=10 and n=20 . Generally, error inflation tends to increase as the number of hypotheses grows. The FWER appears to stabilize for moderate sample sizes of around n=200 . Due to the simulation design, power is unaffected by sample size and depends solely on the number of hypotheses. In Figure S.4, in which Z -scores were generated based on the skew-normal distribution, we observe similar results regarding type I error. Specifically, there is no indication of substantial error inflation, even though the theoretical assumptions have been violated. Overall, the procedures for asymptotic error control seem to work well, even with a finite sample size. One illustrative explanation is that using consistent weights, or more specifically the low intensity bootstrap, in a sense reduces the degree of dependence within the information used to construct the test levels, so that the resulting procedures are comparatively more robust to correlations within the p -values.

### 4.3. Platform trial

We follow the set-up described by Robertson et al.^6^ Let $T_{1},\ldots ,T_{N}$ be treatments that enter the platform trial at time points $t_{1},\ldots ,t_{N}$ , respectively. Throughout the trial, patients are allocated at a uniform rate to the active treatment arms and the common control arm C . The responses are given as Gaussian random variables $X_{ij}∼N(\mu_{i},\sigma^{2})$ , i=0,…,N , with known standard deviation σ . The index 0 corresponds to the control patients. A treatment is generated as effective with probability $\pi_{1}$ . We set $\mu_{i}=0$ if $T_{i}$ is ineffective, $\mu_{i}=0.5$ if it is effective, and $\mu_{0}=0$ . For simplicity, we assume that there will be n patients recruited to each treatment arm $T_{i}$ when the according test is eventually performed. Due to the uniform recruitment rate, there will also be n concurrent control patients for each treatment arm. Let $J_{i}$ be the index set of the concurrent control observations for the treatment arm $T_{i}$ . The test statistic for the i -th test is of the form

$$Z_{i}=\sigma^{-1}(n/2{)}^{1/2}\left\{{\bar{X}}_{i}-{\bar{X}}_{0}^{\left(i\right)}\right\},$$

where ${\bar{X}}_{i}=n^{-1}\sum_{j=1}^{n}X_{ij}$ and ${\bar{X}}_{0}^{\left(i\right)}=n^{-1}\sum_{j\in J_{i}}X_{0j}$ . We assume that per unit time there will be 10 patients allocated to all active treatment arms and the control arm. The time point when the treatment $T_{i}$ enters the trial is defined by $t_{i}=2(i-1)$ . Similar to the previous setting, the random variables $\xi_{i,n}$ are calculated via a parametric low intensity bootstrap in the treatment group and the control group, respectively. We choose α=0.05 , σ=1 , and n=100 , that is, every treatment arm stays in the trial for 10 units of time. For the number of overall treatments, we consider N∈{30,50,100} .

**Figure 5.**
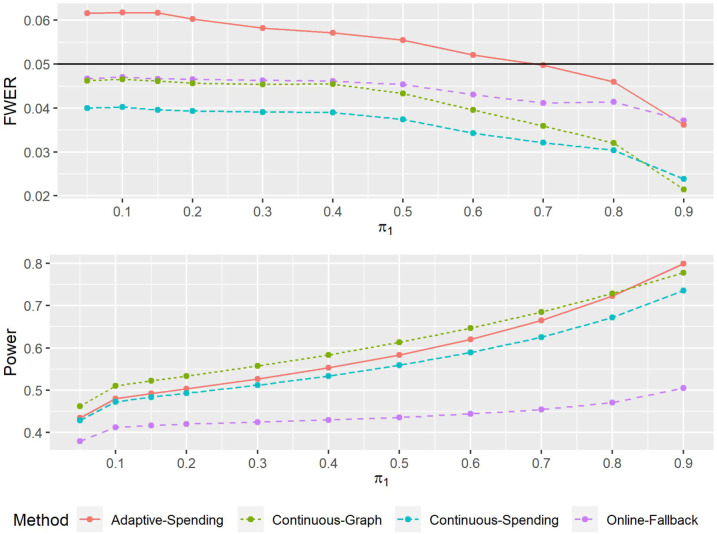
Estimated FWER and power in a platform trial scenario with N=50 and target level α=0.05 plotted over the proportion of false null hypotheses $\pi_{1}$ .

As can be seen in Figure 5, all procedures besides Adaptive-Spending control the FWER. The latter exhibits moderate error inflation, when $\pi_{1}$ is smaller than 0.6 . It is notable that the procedures (especially Continuous-Graph and Continuous-Spending) become quite conservative when the proportion of true alternatives is high. One possible reason for this is, that the number of actually tested hypotheses in this set-up is comparatively small, and a considerable proportion of the regained test level is saved for future hypotheses. This might be counteracted by a different choice of the hyperparameters $\left(\gamma_{i}),(g_{j,i}\right)$ , and f . As in the first setting, we further see that Adaptive-Spending and the Continuous-Graph are the most powerful procedures, after that comes Continuous-Spending and Online-Fallback is again the least powerful method by far.

## 5. Discussion

Existing advanced online multiple testing procedures only guarantee FWER control under the assumption of either independent or, at most, locally dependent p -values. In practice, independence is an unrealistic assumption, and even methods that work for local dependence cannot be applied in some situations, for example, when the dependence of p -values cannot be linked to the intervening time alone or when there is little or no information about the dependency structure at all. In this work we proposed a way to derive online procedures that asymptotically control the FWER, regardless of the underlying dependency structure. To this end, we established general rules for the derivation of new procedures and gave concrete examples. Moreover, we showed how to obtain further improvements of those procedures by using the online closure principle. In a simulation study, we showed that the asymptotic results are also satisfactory for moderate finite sample sizes.

While our simulations indicate that the Continuous-Graph method generally maintains the FWER close to the nominal level and offers higher statistical power than the other evaluated methods, its performance may vary under different data-generating processes or testing scenarios. Rather than advocating for its universal application across all study designs, we recommend that researchers conduct tailored simulation studies to assess each method’s suitability for their specific context, including calibrating relevant hyperparameters. We hope that the provided source code will support such evaluations.

It is worth noting that all of the proposed procedures, except for the closed procedures in Section 3.5, effectively control the expected number of false rejections. So they can as well be used to obtain control of the k -FWER, that is, the probability of making at least k false rejections, at target level α by choosing the nominal level for the procedures as kα .^7^ It might be worthwhile to investigate whether this new approach of dealing with dependency can be applied to further error rates, as for example the (marginal) false discovery rate.

One current shortcoming is that, although the most powerful procedures developed for the case of independent (or locally dependent) p -values use a combination of adaptivity and discarding, only the concept of adaptivity could be transferred to this new setting so far. This has the consequence that the proposed procedures for dependent p -values will lose power when the p -values are conservative, that is, being stochastically larger than a standard uniform random variable. Interestingly, Robertson et al.^6^ did not detect any error inflation of the ADDIS-Spending procedure, which grants formal FWER control only for independent p -values, while we saw in Section 4 that the Adaptive-Spending exceeds the nominal test level in a similar simulation set-up. So, it seems that the additional discarding not only improves the power but also somehow helps to maintain the nominal test level in this case. It might be interesting to investigate how this would behave if the p -values were negatively dependent. In any case, it would be desirable to incorporate any form of discarding or another method for dealing with conservative p -values into the new framework.

Another task for the future would be the extension to more general test set-ups, as only one-sided 1-sample and 2-sample hypothesis tests have been considered in this work. Similar resampling schemes can probably be used to obtain consistent weights in different situations, such as regression models. Moreover, although the proposed approach can, in principle, be applied to various data distributions, we only provide examples for constructing consistent weights specifically for normally distributed data. While this may be adequate in practice for large sample sizes, where the normal approximation is reasonable, alternative strategies for weight construction may be required in settings with heavily skewed or non-normal data.

In addition, there is still interest in developing more efficient methods for testing an initially unknown but finite number of hypotheses. Improvements could be expected here if the design of the procedures takes into account that ultimately, only finitely many hypotheses are tested.

## Supplemental Material

sj-pdf-1-smm-10.1177_09622802261461776 - Supplemental material for Asymptotic online FWER control for dependent test statistics Supplemental material, sj-pdf-1-smm-10.1177_09622802261461776 for Asymptotic online FWER control for dependent test statistics by Vincent Jankovic, Lasse Fischer and Werner Brannath in Statistical Methods in Medical Research

sj-zip-2-smm-10.1177_09622802261461776 - Supplemental material for Asymptotic online FWER control for dependent test statistics Supplemental material, sj-zip-2-smm-10.1177_09622802261461776 for Asymptotic online FWER control for dependent test statistics by Vincent Jankovic, Lasse Fischer and Werner Brannath in Statistical Methods in Medical Research
