# Supplementary material for: Asymptotic online FWER control for dependent test statistics
Source: Stat Methods Med Res. 2026 Jul 2;35(8):1693–708. doi: 10.1177/09622802261461776 (PMC13478442; doi:10.1177/09622802261461776)
Supplement: Supplementary file 1 [file sj-pdf-1-smm-10.1177_09622802261461776.pdf]

Supplementary Material for "Asymptotic online FWER control for dependent test statistics"

Deferred proofs and derivations

Proof of Theorem 3.11. As in the proof of Theorem 3.10, we will effectively show that $\mathbb{E}(V_n(N)) \leq \alpha$ and thus, in particular, the familywise error rate is controlled.

By definition, it holds that

$$\begin{aligned}\mathbb{E}(\xi_{i,n_i}) &= \mathbb{E}\left[\mathbb{P}\{P^*_{i,m(n_i)} > \lambda_i \mid \mathcal{X}_i\}\right] \\ &= \mathbb{P}\{\hat{\theta}^*_{i,m(n_i)} \leq s_{i,m(n_i)}\Phi^{-1}(1-\lambda_i)\}.\end{aligned}$$

Under the null hypothesis, the unconditional distribution of $\hat{\theta}^*_{i,m(n_i)}$ is a normal distribution with expectation 0 and variance $s^2_{i,n_i} + s^2_{i,m(n_i)}$. Since λ_i is assumed to be larger or equal than 0.5 (and thus $\Phi^{-1}(1-\lambda_i) \leq 0$), it holds that $\mathbb{E}(\xi_{i,n_i}) \geq 1 - \lambda_i$ for all $i \geq 1$. This implies $\mathbb{E}(\frac{\xi_{i,n_i}}{1-\lambda_i}|\mathcal{F}^{i-1}) \geq 1$ almost surely, because ξ_{i,n_i} is independent of $\mathcal{F}^{i-1} := \sigma(\mathcal{X}_1, \ldots, \mathcal{X}_{i-1})$.

It follows that

$$\begin{aligned}\mathbb{E}(V_n(N)) &= \sum_{i\in\mathcal{H}^0(N)} \mathbb{E}\{\mathbb{E}(\mathbb{1}\{P_{i,n_i} \leq \alpha_{i,n}\}|\mathcal{F}^{i-1})\} \\ &\leq \sum_{i\in\mathcal{H}^0(N)} \mathbb{E}(\alpha_{i,n}) \\ &\leq \sum_{i\in\mathcal{H}^0(N)} \mathbb{E}\left\{\alpha_{i,n}\mathbb{E}\left(\frac{\xi_{i,n_i}}{1-\lambda_i}|\mathcal{F}^{i-1}\right)\right\} \\ &= \sum_{i\in\mathcal{H}^0(N)} \mathbb{E}\left(\alpha_{i,n_i}\frac{\xi_{i,n_i}}{1-\lambda_i}\right) \\ &\leq \sum_{i\in\mathbb{N}} \mathbb{E}\left(\alpha_{i,n_i}\frac{\xi_{i,n_i}}{1-\lambda_i}\right) \\ &\leq \alpha,\end{aligned}$$

where the first inequality holds due to the independence assumption and in the second equality it is used that $\alpha_{i,n}$ is measurable with respect to $\mathcal{F}^{i-1}$. □

Proof that the procedure from Example 3.12 fulfills condition (6). By definition, we have $\alpha_{1,n} = \Pi_1(1-\lambda_1)\alpha$, and since $\Pi_1, \xi_{1,n} \in [0,1]$, it follows that $\frac{\alpha_{1,n}}{1-\lambda_1}\xi_{1,n} = \Pi_1\xi_{1,n}\alpha \leq \alpha$. Now, assume that for some fixed $j \in \mathbb{N}$,

$$\sum_{i=1}^{j} \frac{\alpha_{i,n}}{1-\lambda_i}\xi_{i,n} \leq \alpha. \tag{S.1}$$

Then, we have

$$\begin{aligned}\sum_{i=1}^{j+1} \frac{\alpha_{i,n}}{1-\lambda_i}\xi_{i,n} &= \frac{\alpha_{j+1,n}}{1-\lambda_{j+1}}\xi_{j+1,n} + \sum_{i=1}^{j} \frac{\alpha_{i,n}}{1-\lambda_i}\xi_{i,n} \\ &= \Pi_{j+1}\xi_{j+1,n}(\alpha - \sum_{i=1}^{j} \frac{\alpha_{i,n}}{1-\lambda_i}\xi_{i,n}) + \sum_{i=1}^{j} \frac{\alpha_{i,n}}{1-\lambda_i}\xi_{i,n} \\ &\leq \alpha,\end{aligned}$$

where the inequality follows from (S.1) and the fact that $\Pi_{j+1}, \xi_{j+1,n} \in [0,1]$. By induction, we conclude that (6) holds for any $N \in \mathbb{N}$. □

Proof that the continuous Adaptive-Graph fulfills condition (6). Since no asymptotic arguments are required within this proof, we omit the index n throughout. We follow the corresponding proof for the ADDIS-Graph.[1] Let $(\alpha_i)_{i\in\mathbb{N}}$ be the test levels of the continuous Adaptive-Graph as defined in (9). We need to show that for every $i \in \mathbb{N}$ it holds that

$$\sum_{j=1}^{i} \frac{\alpha_j}{1-\lambda_j}\xi_j \leq \alpha.$$

We fix an $i \in \mathbb{N}$ and define $U_j = 1 - \xi_j$ for $j \in \{1, \ldots, i\}$. Then the claim is equivalent to

$$F_i(U_{1:i}) = \sum_{j=1}^{i} \left\{ \alpha\gamma_j + \sum_{k=1}^{j-1} g_{k,j}U_k\alpha_k(U_{1:k-1})(1-\lambda_k)^{-1} \right\} (1-U_j) \leq \alpha \tag{S.2}$$

for any $U_{1:i} = (U_1, \ldots, U_i)^T \in [0,1]^i$. First, consider the special case $U^0_{1:i} = (0, \ldots, 0)^T \in [0,1]^i$, which obviously satisfies (S.2), since $F_i(U^0_{1:i}) =$

$\sum_{j=1}^{i} \alpha\gamma_j \leq \alpha$. Now, let $U_{1:i} = (U_1, \dots, U_i)^T \in [0,1]^i$ be arbitrary but fixed. The plan is to show that $F_i(U_{1:i}) \leq F_i(U^0_{1:i})$, which would conclude the proof. Without loss of generality, we assume that $U_j > 0$ for at least one $j \in \{1, \dots, i\}$ as we have already dealt with the case of all entries being zero. Furthermore, define $l = \max\{j \in \{1, \dots, i\} : U_j > 0\}$ and consider $U^l_{1:i} = (U^l_1, \dots, U^l_i)^T$, where $U^l_j = U_j$ if $j \neq l$ and $U^l_l = 0$. For ease of notation we write from now on α_j and α^l_j instead of $\alpha_j(U_{1:j-1})$ and $\alpha_j(U^l_{1:j-1})$, respectively. By the definition of l and $U^l_{1:i}$, for every $j \leq i$ we have that $U^l_j = 0$ for $j \geq l$, $U_j = 0$ for $j \geq l+1$ and $\alpha^l_j = \alpha_j$ for $j \leq l$. Thus, we obtain

$$
\begin{aligned}
&F_i(U^l_{1:i}) - F_i(U_{1:i}) \\
=&\sum_{j=1}^{i} \alpha\gamma_j(U_j - U^l_j) + \sum_{j=1}^{i} \left\{ \sum_{k=1}^{j-1} g_{k,j} U^l_k \alpha^l_k (1-\lambda_k)^{-1} \right\} (1 - U^l_j) \\
&\quad - \sum_{j=1}^{i} \left\{ \sum_{k=1}^{j-1} g_{k,j} U_k \alpha_k (1-\lambda_k)^{-1} \right\} (1 - U_j) \\
=&\alpha\gamma_l U_l + \sum_{k=1}^{l-1} g_{k,l} U^l_k \alpha^l_k (1-\lambda_k)^{-1} - (1-U_l) \sum_{k=1}^{l-1} g_{k,l} U_k \alpha_k (1-\lambda_k)^{-1} \\
&\quad + \sum_{j=l+1}^{i} \left\{ \sum_{k=1}^{j-1} g_{k,j} U^l_k \alpha^l_k (1-\lambda_k)^{-1} \right\} - \sum_{j=l+1}^{i} \left\{ \sum_{k=1}^{j-1} g_{k,j} U_k \alpha_k (1-\lambda_k)^{-1} \right\} \\
=&\alpha\gamma_l U_l + U_l \sum_{k=1}^{l-1} g_{k,l} U_k \alpha_k (1-\lambda_k)^{-1} - \sum_{j=l+1}^{i} g_{l,j} U_l \alpha_l (1-\lambda_l)^{-1} \\
\geq& U_l \left\{ \alpha\gamma_l + \sum_{k=1}^{l-1} g_{k,l} U_k \alpha_k (1-\lambda_k)^{-1} - \alpha_l (1-\lambda_l)^{-1} \right\} \stackrel{(9)}{=} 0,
\end{aligned}
$$

where the inequality holds, because $\sum_{j>l} g_{l,j} \leq 1$ and $U_l, \alpha_l, (1-\lambda_l)^{-1} \geq 0$. So, we have $F_i(U_{1:i}) \leq F_i(U^l_{1:i})$. Iterating this process, it follows that $F_i(U_{1:i}) \leq F_i(U^0_{1:i})$, which completes the proof. □

Proof that the continuous Adaptive-Graph generalizes the procedure in Example 3.12. Let $\lambda_i, \Pi \in (0,1)$, $\gamma_i = \Pi(1-\Pi)^{i-1}, i \in \mathbb{N}$, and $g_{j,i} = \gamma_{i-j}$ for all $j \in \mathbb{N}, i > j$. Further, let $\alpha^{\text{Graph}}_{i,n}$ and $\tilde{\alpha}_{i,n}$ denote the test levels of the continuous Adaptive-Graph (9) and the geometric spending algorithm (7), respectively. We give a proof by induction. By definition it holds that

$$\alpha^{\text{Graph}}_{1,n} = (1-\lambda_1)\gamma_1\alpha = (1-\lambda_1)\Pi\alpha = \tilde{\alpha}_{1,n}.$$

Now assume that $\alpha_{i,n}^{\text{Graph}} = \tilde{\alpha}_{i,n}$ holds for an $i \in \mathbb{N}$. Then,

$$
\begin{aligned}
&\alpha_{i+1,n}^{\text{Graph}} \\
=&(1-\lambda_{i+1})\left\{\gamma_{i+1}\alpha + \sum_{j=1}^{i}\gamma_{i-j+1}(1-\xi_{j,n})\frac{\alpha_{j,n}^{\text{Graph}}}{1-\lambda_j}\right\} \\
=&(1-\lambda_{i+1}) \\
&\quad\left\{(1-\Pi)\gamma_i\alpha + \Pi(1-\xi_{i,n})\frac{\alpha_{i,n}^{\text{Graph}}}{1-\lambda_i} + (1-\Pi)\sum_{j=1}^{i-1}\gamma_{i-j}(1-\xi_{j,n})\frac{\alpha_{j,n}^{\text{Graph}}}{1-\lambda_j}\right\} \\
=&(1-\lambda_{i+1})\left\{\Pi(1-\xi_{i,n})\frac{\alpha_{i,n}^{\text{Graph}}}{1-\lambda_i} + (1-\Pi)\frac{\alpha_{i,n}^{\text{Graph}}}{1-\lambda_i}\right\} \\
=&(1-\lambda_{i+1})\frac{\alpha_{i,n}^{\text{Graph}}}{1-\lambda_i}(1-\Pi\xi_{i,n}) \\
=&(1-\lambda_{i+1})\frac{\tilde{\alpha}_{i,n}}{1-\lambda_i}(1-\Pi\xi_{i,n}) \\
\overset{(8)}{=}&\tilde{\alpha}_{i+1,n}.
\end{aligned}
$$

□

Computation of the constant for the interpolation procedure (10). We compute

$$
\begin{aligned}
\int_1^\infty f_\gamma(x)dx &= \sum_{k=1}^{\infty}\int_k^{k+1} f_\gamma(x)dx \\
&= \sum_{k=1}^{\infty}\int_k^{k+1}\{\gamma_k + (x-k)(\gamma_{k+1}-\gamma_k)\}\,dx \\
&= \sum_{k=1}^{\infty}\left\{\gamma_k + \int_0^1 x(\gamma_{k+1}-\gamma_k)dx\right\} \\
&= \frac{1}{2}\left(\sum_{k=1}^{\infty}\gamma_k + \sum_{k=2}^{\infty}\gamma_k\right) \\
&= 1 - \frac{\gamma_1}{2}.
\end{aligned}
$$

Since $f_\gamma(1) = \gamma_1$, we have $s = (1-\lambda)f_\gamma(1) + \int_1^\infty f_\gamma(x)dx = 1 + \gamma_1(1/2 - \lambda)$.

Proof of Lemma 3.14. Let $j_1 < \cdots < j_k$ denote the indices corresponding to the true null hypotheses among the first N hypotheses and let $0 < \varepsilon < 1 - \lambda$. Due to (5) there exists an $n_0 \in \mathbb{N}$ such that for all $n \geq n_0$ we have $\mathbb{P}(1 - \lambda - \xi_{i,n} \geq \varepsilon) \leq \varepsilon/k$ for all $i \in \mathcal{H}^0(N)$. Setting $S_i := \{i, \dots k\}$, for the test levels defined in (10) it holds that

$$
\begin{aligned}
&\mathbb{E}\left(\sum_{i\in S_1} \alpha_{j_i,n}\right) \\
&\leq \mathbb{E}\left\{\frac{\alpha}{s}\sum_{i\in S_1}(1-\lambda)f\left(1+\sum_{l<i}\xi_{j_l,n}\right)\right\} \\
&= \frac{\alpha}{s}\mathbb{E}\left\{(1-\lambda)f(1) + \sum_{i\in S_2}\mathbb{1}\{\xi_{j_{i-1},n} > 1-\lambda-\varepsilon\}(1-\lambda)f\left(1+\sum_{l<i}\xi_{j_l,n}\right)\right\} \\
&\quad + \frac{\alpha}{s}\mathbb{E}\left\{\sum_{i\in S_2}\mathbb{1}\{\xi_{j_{i-1},n} \leq 1-\lambda-\varepsilon\}(1-\lambda)f\left(1+\sum_{l<i}\xi_{j_l,n}\right)\right\} \\
&\leq \frac{\alpha}{s}\mathbb{E}\left\{(1-\lambda)f(1) + \sum_{i\in S_2}\mathbb{1}\{\xi_{j_{i-1},n} > 1-\lambda-\varepsilon\}\frac{1-\lambda}{\xi_{j_{i-1},n}}\int_{1+\sum_{l<i-1}\xi_{j_l,n}}^{1+\sum_{l<i}\xi_{j_l,n}} f(x)dx\right\} \\
&\quad + \frac{\alpha}{s}(1-\lambda)f(1)\sum_{i\in S_2}\mathbb{P}(\xi_{j_{i-1},n} \leq 1-\lambda-\varepsilon) \\
&\leq \frac{\alpha}{s}\mathbb{E}\left\{(1-\lambda)f(1) + \sum_{i\in S_2}\frac{1-\lambda}{1-\lambda-\varepsilon}\int_{1+\sum_{l<i-1}\xi_{j_l,n}}^{1+\sum_{l<i}\xi_{j_l,n}} f(x)dx\right\} \\
&\quad + \alpha\sum_{i\in S_2}\frac{\varepsilon}{k} \\
&\leq \frac{\alpha}{s}\mathbb{E}\left\{\frac{1-\lambda}{1-\lambda-\varepsilon}(1-\lambda)f(1) + \sum_{i\in S_2}\frac{1-\lambda}{1-\lambda-\varepsilon}\int_{1+\sum_{l<i-1}\xi_{j_l,n}}^{1+\sum_{l<i}\xi_{j_l,n}} f(x)dx\right\} + \varepsilon \\
&\leq \frac{\alpha(1-\lambda)}{s(1-\lambda-\varepsilon)}\mathbb{E}\left\{(1-\lambda)f(1) + \int_1^\infty f(x)dx\right\} + \varepsilon \\
&= \alpha\frac{1-\lambda}{1-\lambda-\varepsilon} + \varepsilon.
\end{aligned}
$$

Here, in the first and second inequality it is used, that f is non-increasing. As ε was arbitrary, we conclude $\lim_{n\to\infty}\mathbb{E}\left(\sum_{i\in S_1}\alpha_{j_i,n}\right) \leq \alpha$. Since $\alpha_{i,n} \to \alpha_i$ in probability, we also have that $\sum_{i\in\mathcal{H}^0(N)}\alpha_{i,n} \to \sum_{i\in\mathcal{H}^0(N)}\alpha_i$ in probability.

The dominated convergence theorem for convergence in probability[2] plus the fact that $\sum_{i\in\mathcal{H}^0(N)} \alpha_{i,n} \leq k$ then yield

$$\alpha \geq \mathbb{E}\left(\sum_{i\in\mathcal{H}^0(N)} \alpha_i\right) = \sum_{i\in\mathcal{H}^0(N)} \alpha_i.$$

This concludes the proof. □

Derivation of the closed procedures (13) and (14). Based on the test levels (9), for every $I \subseteq \mathbb{N}$ we consider the intersection test that rejects the intersection hypothesis $H_I = \bigcap_{i\in I} H_i$ if and only if for at least one $i \in I$ it holds that

$$P_{i,n} \leq \alpha_{i,n}^I = (1-\lambda_i)\left\{\alpha\gamma_i + \sum_{j\in I, j<i} g_{j,i}(1-\xi_{j,n})\frac{\alpha_{j,n}^I}{1-\lambda_j} + \sum_{j\notin I, j<i} g_{j,i}\frac{\alpha_{j,n}^{(I\cup\{j\})}}{1-\lambda_j}\right\}.$$

Note that $\alpha_{1,n}^I = (1-\lambda_1)\alpha\gamma_1$ if $1 \in I$ and all further test levels are defined recursively. It is easy to see, that this defines a predictable and consonant intersection test,[3] since the test levels depend only on previous indices and for any $i \in I$ it holds that $\alpha_{i,n}^I \leq \alpha_{i,n}^J$ for all $J \subseteq I$ with $i \in J$. Moreover, it is an (asymptotical) level α test, as it fulfills (6) (with the summation only taking place over the index set I), because the original procedure (9) does and the case $j \notin I$ can be interpreted as $\xi_{j,n} = 0$. Now, we recursively define $I_1 = \{1\}$ and $I_i = \{j \in \mathbb{N} : j < i, P_{j,n} > \alpha_{j,n}^{I_j}\} \cup \{i\}$ for $i \geq 2$. The short-cut procedure[3] is then given by

$$\alpha_{i,n} = \alpha_{i,n}^{I_i} = (1-\lambda_i)\left(\alpha\gamma_i + \sum_{j<i} g_{j,i}\max\{1-\xi_{j,n}, R_{j,n}\}\frac{\alpha_{j,n}}{1-\lambda_j}\right).$$

Analogous, the test levels for the intersection tests based on the procedure (10) are defined as

$$\alpha_{i,n}^I = \alpha\frac{1-\lambda}{s} f\left(1 + \sum_{j\in I, j<i} \xi_{j,n}\right).$$

Again, it is easy to verify that these intersection tests are predictable and consonant. Furthermore, they are asymptotical level α tests, as can be

seen by slightly modifying the proof of Lemma 3.14. With I_i as above, the short-cut procedure is defined as

$$\alpha_{i,n} = \alpha_{i,n}^{I_i} = \alpha \frac{1-\lambda}{s} f \left\{ 1 + \sum_{j<i} \xi_{j,n}(1 - R_{j,n}) \right\}.$$

Obtaining consistent weights in a 2-sample design. Assume that the observed data $\mathcal{X}$ consists of independent random variables $X_1, \ldots, X_{n_1} \sim \mathcal{N}(\mu^X, 1), Y_1, \ldots, Y_{n_2} \sim \mathcal{N}(\mu^Y, 1)$ and we want to test the hypothesis $H : \mu^X - \mu^Y \leq 0$ versus the alternative $H' : \mu^X - \mu^Y > 0$. Here, $n_1 = n_1(n)$ and $n_2 = n_2(n)$ are the sample sizes for the respective groups, which depend implicitly on the overall asymptotic parameter n. The test statistic and the corresponding one-sided p-value are $Z_n = (1/n_1 + 1/n_2)^{-1/2}(\overline{X}_{n_1} - \overline{Y}_{n_2})$ and $P_n = 1-\Phi(Z_n)$, respectively. Similar to Example 3.3 consider the parametric bootstrap variables $X_1^*, \ldots, X_{m(n_1)}^*, Y_1^*, \ldots, Y_{m(n_2)}^*$, which are independently drawn from the distributions $\mathcal{N}(\overline{X}_{n_1}, 1)$ and $\mathcal{N}(\overline{Y}_{n_2}, 1)$, respectively. As before, $m : \mathbb{N} \to \mathbb{N}$ is a non-decreasing map such that $m(n) \to \infty$ and $m(n)/n \to 0$ as $n \to \infty$. Furthermore, we construct the (low intensity) bootstrap test statistic

$$Z_{m(n)}^* = \{1/m(n_1)+1/m(n_2)\}^{-1/2} \left\{ m(n_1)^{-1} \sum_{j=1}^{m(n_1)} X_j^* - m(n_2)^{-1} \sum_{j=1}^{m(n_2)} Y_j^* \right\},$$

whose conditional distribution is a normal distribution with variance 1 and mean

$$\frac{(1/n_1 + 1/n_2)^{1/2}}{\{1/m(n_1) + 1/m(n_2)\}^{1/2}} Z_n.$$

Moreover, we define the low intensity bootstrap version of the p-value P_n by $P_{m(n)}^* = 1 - \Phi\{Z_{m(n)}^*\}$. For any $\lambda \in (0, 1)$, we can now define the consistent weights by $\xi_n = \mathbb{P}\{P_{m(n)}^* > \lambda | \mathcal{X}\}$. The consistency condition (5) can be shown completely analogous to Example 3.3 if $n_1 = n_2$. In general, it is necessary that the groups are at least asymptotically balanced, i.e. $n_1/n_2 \to 1$ as $n \to \infty$.

Further simulation results

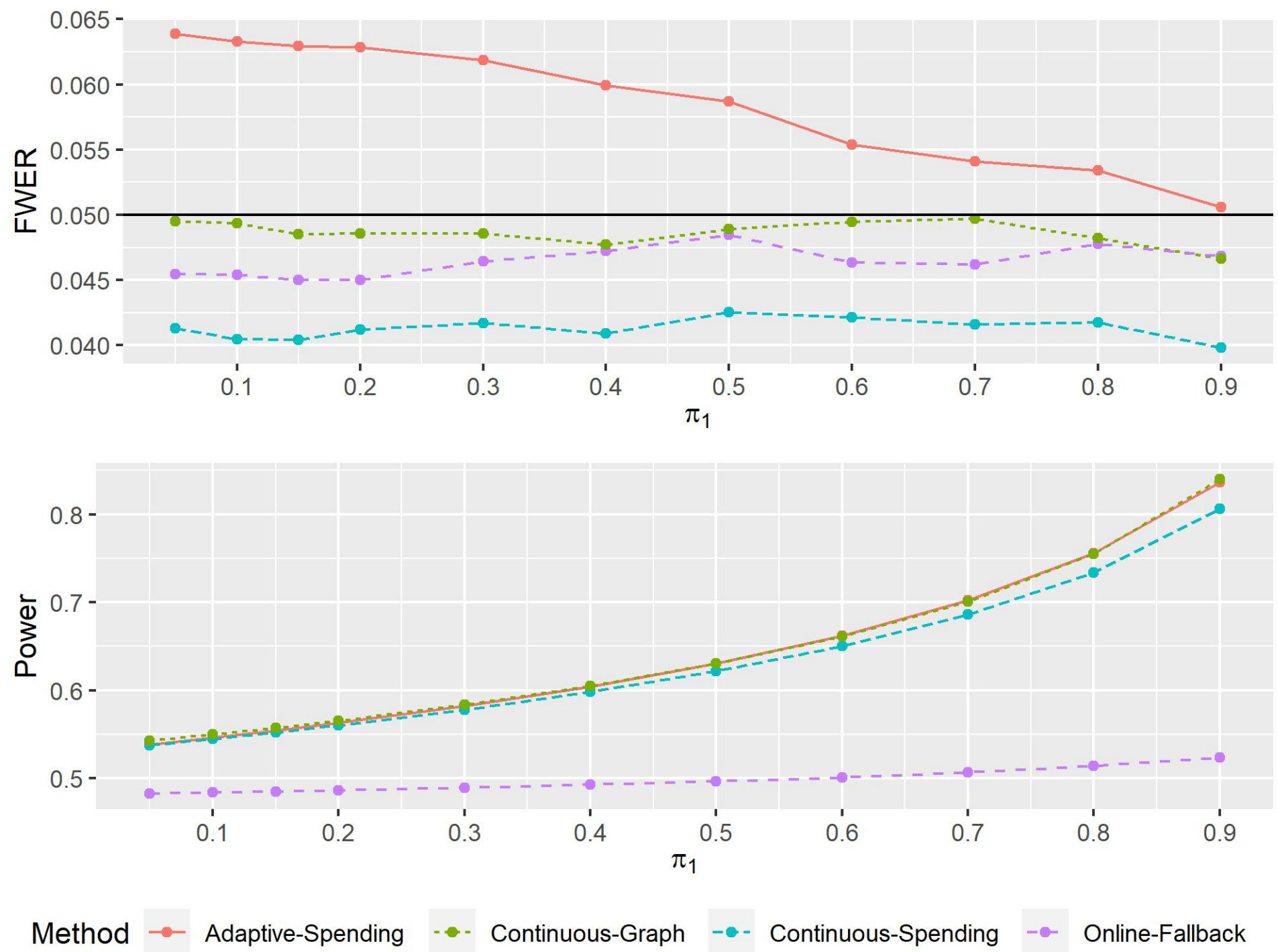

Figure S.1: Estimated FWER and power in an autocorrelation set-up with $N = 1000$, $\rho = 0.5$ and target level $\alpha = 0.05$ plotted over the proportion of false null hypotheses π_1.

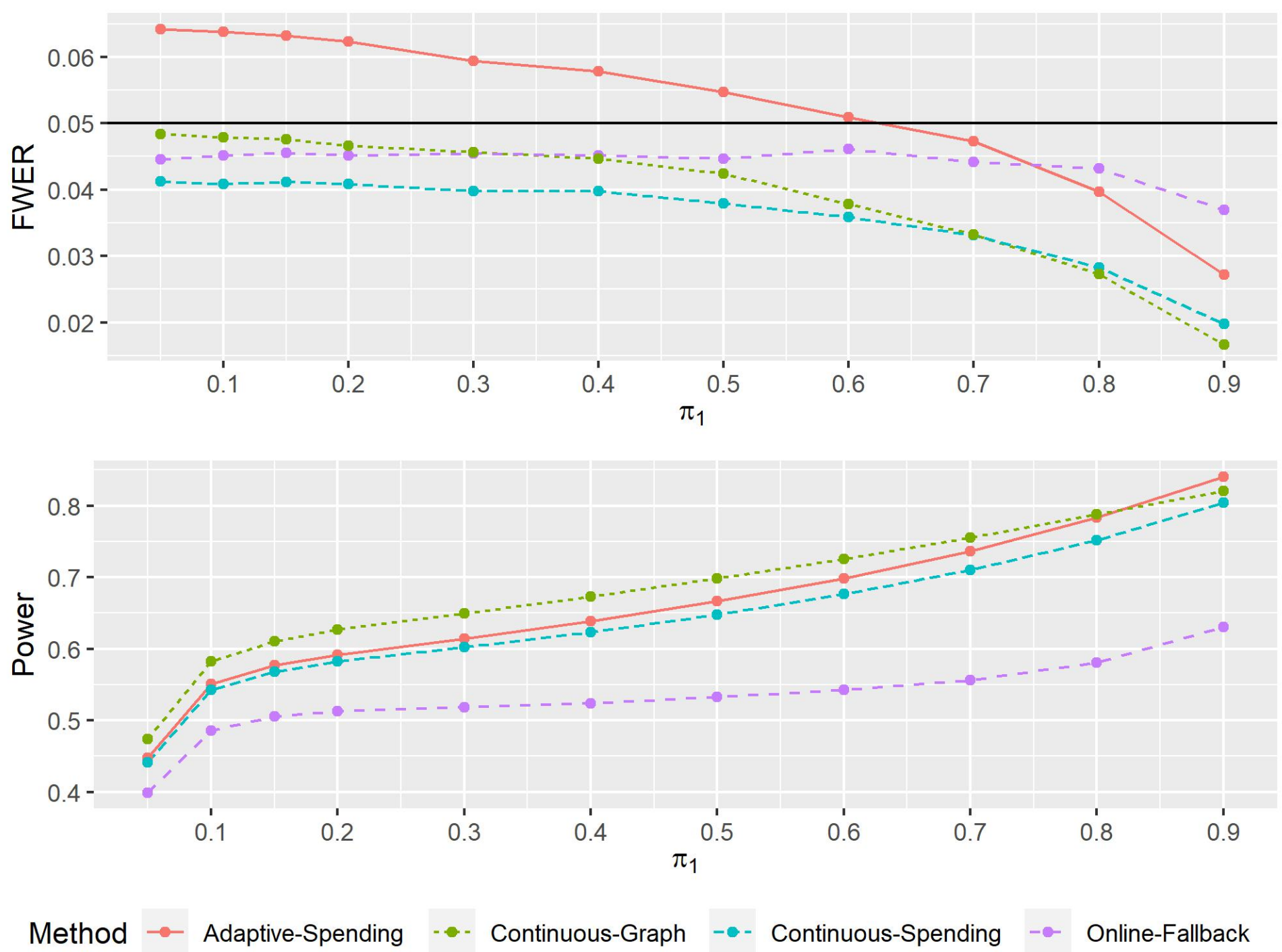

Figure S.2: Estimated FWER and power in a platform trial scenario with $N = 30$ and target level $\alpha = 0.05$ plotted over the proportion of false null hypotheses π_1.

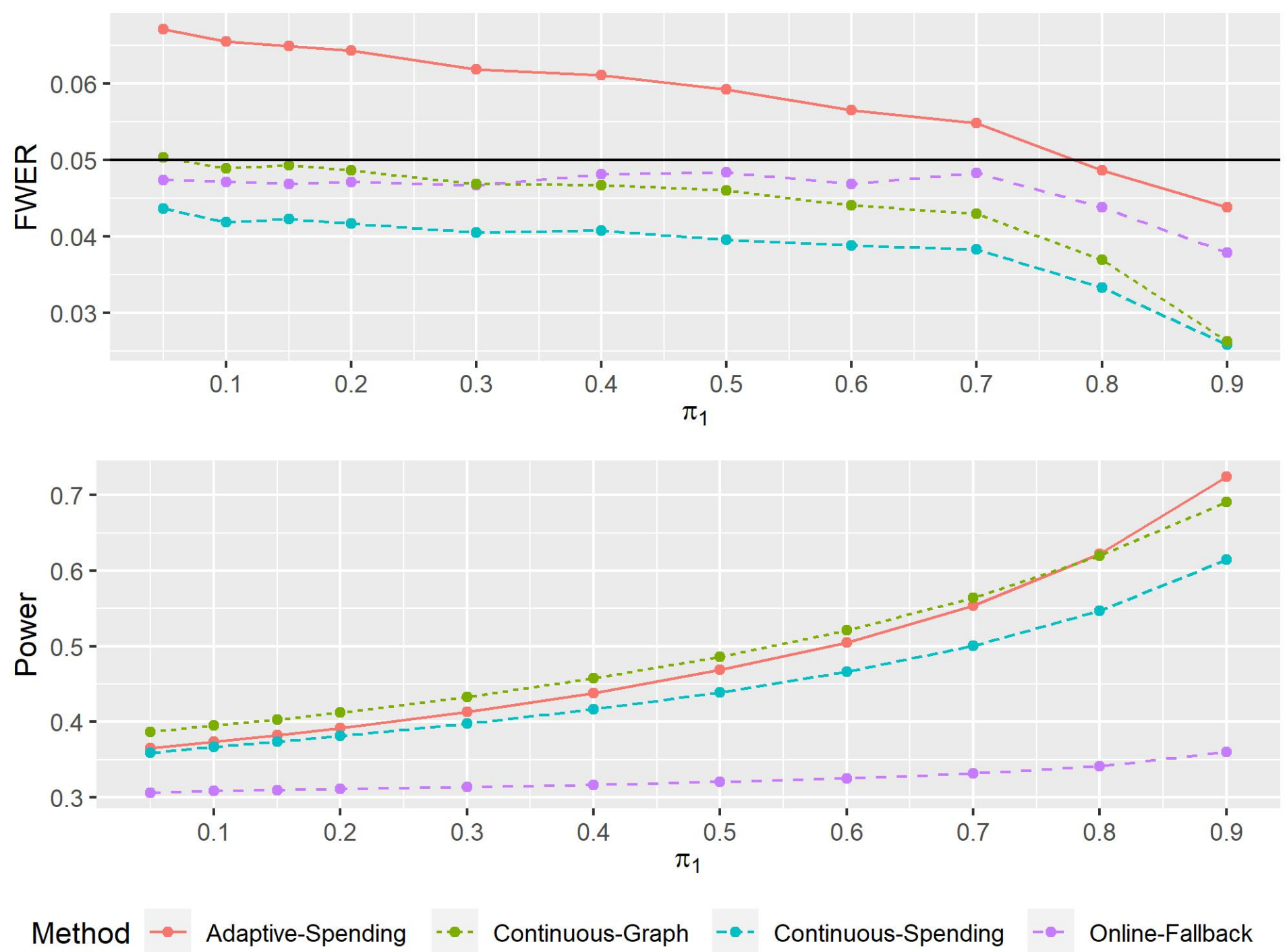

Figure S.3: Estimated FWER and power in a platform trial scenario with $N = 100$ and target level $\alpha = 0.05$ plotted over the proportion of false null hypotheses π_1.

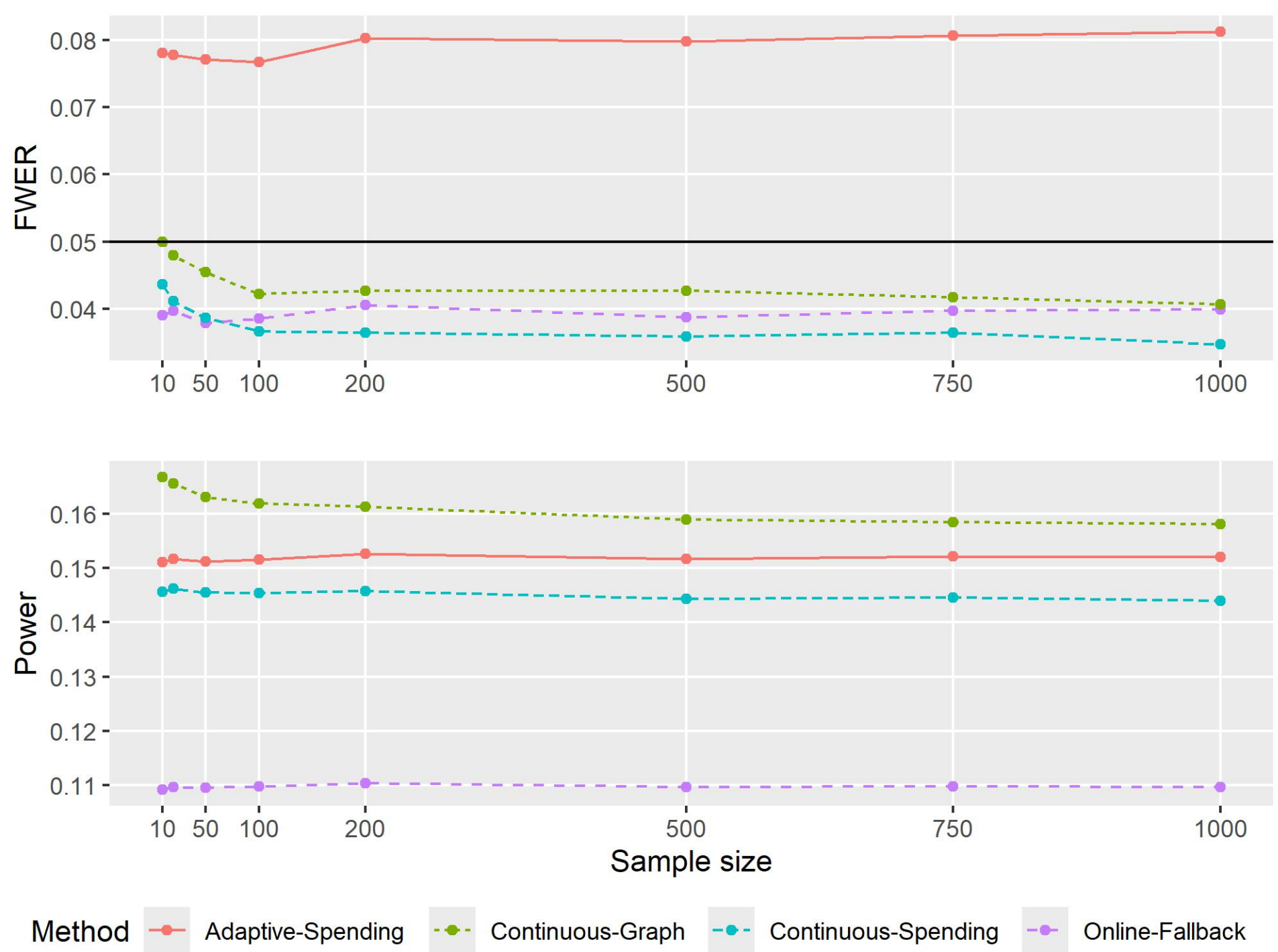

Figure S.4: Estimated FWER and power in an autocorrelation set-up with $N = 500$, $\rho = 0.8$, $\pi_1 = 0.1$, target level $\alpha = 0.05$, where Z-scores have been generated based on a skew-normal distribution with skewness parameter 0.5.

References

[1] Fischer L, Bofill Roig M and Brannath W. An adaptive-discard-graph for online error control. *arXiv preprint arXiv:230111711* 2023; .

[2] Bogachev VI. *Measure theory*, volume 1. Springer, 2007.

[3] Fischer L, Bofill Roig M and Brannath W. The online closure principle. *The Annals of Statistics* 2024; 52(2): 817–841.
